# The Known Unknowns of the Human Dendritic Cell Network

**DOI:** 10.3389/fimmu.2015.00129

**Published:** 2015-03-23

**Authors:** Mélanie Durand, Elodie Segura

**Affiliations:** ^1^Centre de Recherche, Institut Curie, Paris, France; ^2^INSERM U932, Paris, France

**Keywords:** human, dendritic cells, antigen presentation, DC subsets, ontogeny

## Abstract

Dendritic cells (DCs) initiate and orient immune responses and comprise several subsets that display distinct phenotypes and properties. Most of our knowledge of DC subsets biology is based on mouse studies. In the past few years, the alignment of the human DC network with the mouse DC network has been the focus of much attention. Although comparative phenotypic and transcriptomic analysis have shown a high level of homology between mouse and human DC subsets, significant differences in phenotype and function have also been evidenced. Here, we review recent advances in our understanding of the human DC network and discuss some remaining gaps and future challenges of the human DC field.

## Introduction

Dendritic cells (DCs) have long been known to be the most efficient antigen-presenting cells. It is now well established that DCs are a heterogeneous population composed of several subsets that can be distinguished by their phenotype, location, and functional properties (1). Due to their remarkable ability to stimulate T cells, DCs have become in the past decade attractive therapeutic targets. However, most of our knowledge of DC subsets biology was gained from mouse studies, and cross-species differences could hinder the successful translation to humans of major discoveries made in the mouse. In the past few years, a number of studies have tackled the analysis of human DC subsets. In this review, we summarize recent advances and highlight some of the outstanding questions that remain to be addressed.

## How to Define DC Subsets in Humans?

Historically, human DC subsets have been defined based on a small number of phenotypic markers, within the population of MHC class II^+^ lineage-negative cells. In blood, DCs have been divided into two main groups: plasmacytoid DCs (pDC) and “myeloid” or “classical” DCs (cDCs). cDCs can be further separated into two subsets that are usually referred to as BDCA1/CD1c^+^ DCs and BDCA3/CD141^+^ DCs (2). These three DC populations are also found in all lymphoid organs and represent resident DCs (3–6). In skin, liver, lung, and intestine, two main populations of CD1c^+^CD1a^+^ DCs and CD141^+^Clec9A^+^ DCs have been identified (7–12). Tissue DCs can migrate through the lymph to the draining lymph nodes where these migratory DCs display a mature phenotype (4, 13, 14). Additional DC subsets have been described in mucosal tissues: Langerhans cells (LCs) and CD14^+^ DCs (15, 16) in skin and vaginal mucosa, and CD103^−^CD172a^+^ DCs in the intestine (10). Finally, a population of “inflammatory” DCs with a distinct phenotype can also be found in inflamed tissues (17, 18).

Although surface markers are useful for the characterization of DC subsets (Table 1), phenotypic analysis has proven insufficient on its own to define DC subsets. Indeed, some phenotypic markers are not specific of a given DC subset or their expression can change upon activation, potentially leading to misinterpretation. For instance, CD141 is upregulated upon activation on pDC and CD1c^+^ DCs (19) and is also expressed by tissue CD14^+^ DCs (20). Clec9A, which is restricted to CD141^+^ DCs, is downregulated rapidly during DC maturation (21). Another hurdle is the promiscuous expression of some markers on macrophages and monocytes, such as CD14 or CD64. Recently, CD14^+^CD1c^low^ cells in the skin were re-defined as macrophages (22). However, the identity of tissue CD14^+^CD1c^high^ cells remains uncertain, we refer to these cells as CD14^+^ DCs throughout this review.

**Table 1:** **Phenotypic markers for human DC subsets**.

Surface marker	pDC	Blood/resident CD1c DC	Blood/resident CD141 DC	Tissue/migratory CD1c CD1a DC	Tissue/migratory CD141 Clec9A DC	Tissue/migratory CD14 DC	Inflammatory DC	Langerhans cells
HLA-DR	+	+	+	+	+	+	+	+
CD11c	−	++	+	++	+	++	++	+
CD123	+	−	−	−	−	−	−	−
BDCA2/CD303	+	−	−	−	−	−	−	−
BDCA4/CD304	+	−	−	−	−	?	?	−
Clec9A	−	−	+ Immature	−	+	−	−	−
			Low mature	
BDCA3/CD141	−	+ Immature	++	+ Immature	++	+	?	−
	+ Mature	++ Mature		++ Mature	
XCR1	−	−	+	−	+	−	−	−
CX3CR1	?	+ Blood	−	+/−	−	+	?	+/−
		? Lymphoid organs	
BDCA1/CD1c	−	+	−	+	−	+	+	+
Sirp-α/CD172a	−	+	−	+	−	+	+	+
CD11b	−	−Blood	−	+	−	+	+	+
		+ Lymphoid organs	
MR/CD206	−	−	−	+	−	+	++	−
CD14	−	−	−	−	−	+	+	−
FcεRI	−	+	−	?	−	?	+	−
CD1a	−	−	−	+/−	−	−	+	++
CD64	−	+	−	+	−	?	+	?
Langerin/CD207	−	−	−	+/−	−	−	−	+
EpCAM/CD326	−	−	−	−	−	−	−	+
E-cadherin	−	−	−	−	−	−	−	+

*+/−, reported in some tissues. ?, not reported*.

The analysis of key DC properties can help assessing the DC identity of a potential subset. Hallmark properties include dendritic morphology, migratory capacity, and ability to stimulate naive T cells. These properties have been used to distinguish macrophages from DCs in the skin (22, 23) and inflammatory fluids (17), or monocytes from DCs in the blood (24).

Finally, gene expression signatures have emerged from transcriptomic studies and can be a useful tool to confirm DC identity, to assign a population to a known DC subset, or to define a new one. Lineage-negative CD16^+^ blood cells were initially termed CD16^+^ DCs, but transcriptomic analysis showed that they are a subset of monocytes (25). Similarly, 6-sulfo LacNac/Slan^+^ blood cells were termed Slan^+^ DCs, but comparative transcriptomic analysis identified them as a subpopulation of CD16^+^ monocytes (24). Recently, dermal CD14^+^CD1c^low^ cells were found to be closer in their gene expression to macrophages than to DCs (22). Transcriptomic analysis has also been used to assess the proximity of tissue DC subsets with their blood counterparts for skin (8) or intestinal DCs (10).

## What is the Ontogeny of Human DC Subsets?

Addressing human DC ontogeny is challenging, but *in vitro* culture models, clinical observations, and comparative transcriptomic analysis have provided substantial insight. Human DCs are constantly replenished from bone marrow precursors as shown by the replacement of dermal DCs after hematopoietic stem cell transplantation (23) and the loss of blood DCs after bone marrow suppression induced by preparative cytotoxic therapy (22). Patients carrying mutations in *GATA2* or *IRF8* lack all blood DC subsets, consistent with a common origin (26, 27). Several lines of evidence indicate that Flt3-L is required for the generation and/or maintenance of most DC subsets: injection of Flt3-L to human volunteers increases the number of blood DC subsets (28, 29); pDCs, CD1c^+^ DCs, and CD141^+^ DCs equivalents can be derived *in vitro* by culturing CD34^+^ hematopoietic precursors with Flt3-L (30–32); levels of serum Flt3-L are elevated in patients affected by mutations in *GATA2* or *IRF8* (26, 27). The importance of other cytokines in DC differentiation or homeostasis *in vivo* is unclear. Recently, a committed DC progenitor (CDP) has been identified in bone marrow and cord blood, but was absent from adult blood and tonsils (33). In an *in vitro* culture model, these CDP give rise only to pDC and cDCs, via an intermediate precursor restricted to CD1c^+^ DCs and CD141^+^ DCs (29, 33). This pre-cDC is present in adult bone marrow, blood, and tonsils (29). Whether pre-cDC differentiate into cDCs in the blood or lymphoid organs and tissues remains to be addressed.

The ontogeny of migratory DCs also remains to be better characterized. Of note, patients affected with a mutation in *GATA2* retain normal numbers of epidermal LC (27), showing that LC represent a distinct lineage from pDCs and cDCs. The observations that LC remained of donor origin 10 years after hand allograft and that they could proliferate *in situ* indicate that LC can self-renew in tissues (34). In addition, transcriptomic analysis shows that intestinal CD103^−^CD172a^+^ DCs (10) and inflammatory DCs (17) express monocyte gene signatures, suggesting that these DC subsets derive from monocytes rather than a common DC precursor.

Cross-species comparative transcriptomic analysis suggest that pDCs, CD1c^+^ DCs, and CD141^+^ DCs represent distinct *bona fide* lineages, as homologies have been evidenced with the well-defined mouse DC subsets pDCs, CD11b^+^ DCs, and CD8^+^ DCs, respectively (8, 10, 25). Regarding the molecular ontogeny, *in vitro* culture models indicate that the transcription factors E2-2 and Batf3 drive the differentiation of pDCs and CD141^+^ DCs, respectively (35–37). Of note, Batf3 silencing in humanized mice was not sufficient to inhibit CD141^+^ DC differentiation (37), which might be due to molecular compensation by related transcription factors as shown in Batf3-deficient mice (38). It has been proposed that CD1c^+^ DCs depend on IRF4 based on its preferential expression in CD1c^+^ DCs (10, 39), however this remains to be formally proven.

Another unresolved matter is the relationship of blood cDCs and their lymphoid organ and tissue counterparts. It has been suggested that blood CD1c^+^ DCs and CD141^+^ DCs represent a precursor form of cDC subsets (4, 8, 40), but a direct precursor–progeny relationship remains unclear. Consistent with the idea that they are not terminally differentiated, blood CD1c^+^ DCs and CD141^+^ DCs become competent for cross-presentation only after activation, whereas lymphoid organ DCs cross-present without the need for activation (41). Moreover, blood CD1c^+^ DCs retain some plasticity as they can differentiate *ex vivo* into LC-like cells, while tonsil CD1c^+^ DCs cannot (42, 43).

## Is There a Functional Specialization of Human DC Subsets?

### Pathogen recognition

Among the variety of pathogen-recognition receptors, TLR expression by DC subsets (either mRNA or protein expression) has been the most studied. pDC express TLR1, TLR6, TLR7, TLR9, and TLR10, resident CD1c^+^ DCs express TLR1, TLR2, TLR4, TLR5, TLR6, and TLR8, and resident CD141^+^ DCs express TLR1, TLR3, TLR6, TLR8, and TLR10 (3, 5, 44–46). Skin LC express TLR1, TLR2, TLR3, TLR6, TLR7 and vaginal mucosa LC express TLR8 in addition (15, 47, 48), skin and vaginal mucosa CD1a^+^ DCs express TLR6 and TLR8 while the expression of other TLR is less clear, and skin and vaginal mucosa CD14^+^ DCs express TLR1, TLR2, TLR4, TLR6, and TLR8 (15, 49). C-type lectin receptors are also important pathogen-recognition receptors, some of which have been reported to be differentially expressed by DC subsets by transcriptomic analysis (17, 49–52). Receptors whose differential expression among DC subsets has been confirmed at protein level include Clec9A on CD141^+^ DCs, BDCA2/CD303 on pDC, ClecSF14/CD301 on CD1c^+^ DCs, Langerin/CD207 on LC, and Clec10a and LOX-1 on CD14^+^ DCs (5, 19, 49, 53, 54).

Differential expression of pathogen-recognition receptors can confer functional specialization to DC subsets for the response to pathogens (46, 55, 56) or vaccines (57).

Much work remains to be done to characterize the expression pattern of intracellular pathogen-recognition receptors in resident and migratory DC subsets. It has been reported so far that vaginal mucosa and skin LC, CD1a^+^ DCs, and CD14^+^ DCs, all express MDA-5, while only CD14^+^ DCs express RIG-I (15, 49).

### Cytokine secretion

Blood and lymphoid organ pDC have long been known to be the best producers of type I interferon (58–60). CD141^+^ DCs from blood and from humanized mice spleen have also been reported to be the most potent for type I interferon production after TLR3 stimulation (5, 61). More recently, blood and liver CD141^+^ DCs were shown to selectively secrete type III interferon after activation with TLR3 ligand or Hepatitis C virus (56, 62, 63).

Because cytokine secretion by a given DC subset vary depending on the stimulus used (45), it can be difficult to determine *bona fide* specialization for cytokine secretion. Accumulating evidence indicates that blood CD1c^+^ DCs are the best producers of IL-12p70, as shown by stimulation with TLR2, TLR3, and TLR8 ligands (40, 45, 46). Whether CD1c^+^ DCs from tissues are also specialized for IL-12p70 secretion needs to be confirmed. Indeed, no IL-12p70 secretion could be detected after stimulation of skin DC subsets (8) or intestinal CD1c^+^ DCs (11) with several TLR-ligands. Intestinal and lung CD1c^+^ DCs are also the best producers of IL-23 after TLR8 stimulation or *Aspergillus fumigatus* exposure, respectively (11, 39). CD1c^+^ DCs from skin, intestine, and blood are also the most potent producers of IL-10 in response to several TLR-ligands (8, 11, 64). Skin LC and CD1a^+^ DCs have been reported to be better producers of IL-15 than skin CD14^+^ DCs (53, 65), but IL-15 secretion by other DC subsets has not been analyzed yet.

### Cross-presentation and CD8 T-cell responses

Numerous studies have shown that blood and lymphoid organ DC subsets can all cross-present efficiently various forms of antigen (66). Spleen, lymph node, and tonsil CD1c^+^ and CD141^+^ DC subsets are equally potent for cross-presenting soluble antigens, without the need for activation (4, 41, 44). When stimulated with TLR-ligands that can activate both subsets, blood CD1c^+^ and CD141^+^ DCs also display similar efficiency for cross-presentation (40, 44, 67). However, lymphoid organ and activated blood CD141^+^ DCs appear to be more efficient for the cross-presentation of dead cell-derived antigen (5, 41, 68), which might be due to their selective expression of necrotic cell receptors such as Clec9A. Blood CD141^+^ DCs were also more efficient than CD1c^+^ DCs for cross-presentation of antigens delivered to late endocytic compartments via CD205 targeting, but were equally potent after antigen delivery to early endocytic compartments via CD40 (69).

Blood and lymphoid organ pDCs cross-present efficiently soluble (41, 44, 67, 70, 71), viral (71–74), cell-associated antigen (67, 75), or antigen targeted to surface receptors such as CD40, DCIR, CD205, BDCA2/CD303, or CD32 (67, 69, 76).

The ability of tissue DCs to cross-present is less well characterized. Skin CD1a^+^ DCs and LC have been shown to cross-present when purified from skin or skin-draining lymph nodes (4, 77), however, a subsequent study reported that skin CD141^+^Clec9A^+^ DCs are the most efficient for cross-presentation compared to other skin DC subsets (8). Skin LC also cross-present antigen targeted through DCIR (76). Both CD1c^+^ DCs and CD141^+^ DCs from the lung of humanized mice can cross-present (9), but these results need to be confirmed with DCs directly purified from human lung. The cross-presentation capacity of migratory DCs from other tissues and of inflammatory DCs remains to be analyzed. In addition, which DC subsets cross-present *in vivo* in a physiological situation is a challenging question that is still unaddressed.

Skin LC and CD1a^+^ DCs induce the differentiation of cytotoxic T lymphocytes (CTL) more efficiently than skin CD14^+^ DCs, through the secretion of IL-15 (53, 65). Activated LC also express higher levels of CD70, which promotes CTL differentiation (55, 77). Blood-activated CD1c^+^ DCs induce higher expression of granzymes B and K by CTL than activated CD141^+^ DCs, due to the selective secretion of IL-12p70 (40). Whether this specialization also applies to lymphoid organ and tissue CD1c^+^ DCs remains to be confirmed.

### CD4 T cells responses

The vast majority of studies have analyzed the ability of isolated DC subsets to stimulate and polarize allogeneic naive CD4 T cells. Blood, lymph node, or lung CD1c^+^ DCs and CD141^+^ DCs are equally competent for Th1 polarization, either without activation (4, 78) or after exposure to influenza virus (78) or *A. fumigatus* (39). By contrast, blood and lung CD141^+^ DCs have been found to be more potent inducers of Th2 polarization compared to CD1c^+^ DCs, with or without activation, due to the selective expression of OX40-L (78). Lung CD1c^+^ DCs exposed to *A. fumigatus* are more potent than CD141^+^ DCs for Th-17 polarization due to their secretion of IL-23 (39), however both intestinal CD1c^+^ DCs and CD141^+^ DCs are equally able to induce Th-17 polarization (10). Blood pDC can induce Th1 polarization after activation with CD40-L, influenza virus, or Sendai virus (60, 79), but induce Th2 polarization through OX40-L after activation with IL3 (79). Whether these observations also apply to lymphoid organ pDC, and whether pDC can induce Th-17 polarization when adequately activated remains to be addressed.

Skin DC subsets have been proposed to be specialized for CD4 T-cell polarization, LC, and CD1a^+^ DCs being especially potent for Th2 polarization while CD14^+^ DCs mainly induce T follicular helper (Tfh) cells (53). This specialization is conserved after skin DC migration to draining lymph nodes (4). The molecular mechanism underlying this functional specialization remains unclear. Moreover, vaginal mucosa LC and CD1a^+^ DCs preferentially induce Th2 polarization, while vaginal mucosa CD14^+^ DCs are better inducers of Th1 (15). The ability of vaginal mucosa DCs to induce Tfh has not been analyzed. In addition, skin LC have been found to be more potent than other skin DCs for the induction of IL22-secreting CD4 T cells (80, 81), while both vaginal mucosa LC and CD1a^+^ DCs are equally competent (15).

Inflammatory DCs isolated from rheumatoid arthritis synovial fluid and from tumor ascites preferentially induce Th-17 polarization through the secretion of Th-17 polarizing cytokines IL-6, IL-23, TGFβ, and IL1-β (17). Inflammatory DCs from tumor ascites also efficiently stimulate autologous effector CD4 T cells to secrete IL-17 (17). The CD4 T-cell responses induced by inflammatory DCs from other inflammatory environments remain to be investigated.

Finally, several DC subsets were shown to induce Treg: dermal CD14^+^ DCs (20), intestinal CD1c^+^ DCs and CD103^−^CD172a^+^ DCs (10), tonsil pDC activated with IL3 or TLR-ligands (82), and bacteria-exposed skin LC (83). In addition, liver DCs (7) and TLR4-activated oral mucosa DCs (84) were proposed to promote Treg induction through the secretion of IL-10, but whether one subset is more potent for Treg induction has not been analyzed. Blood *Escherichia coli*-activated CD1c^+^ DCs have also been proposed to inhibit CD4 T-cell proliferation through IL-10 secretion (64). In addition, skin LC, but not dermal CD1a^+^ DCs and CD14^+^ DCs, have been shown to stimulate the proliferation of autologous skin-resident memory Treg (85).

**Figure 1:**
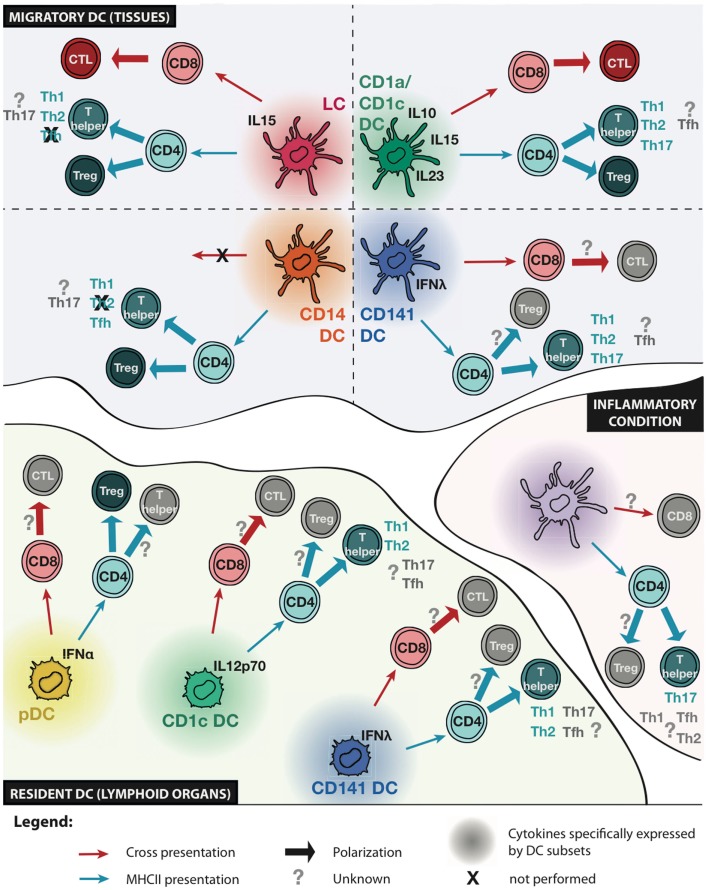
**Functional specialization of human DC subsets**. Schematic representation of known and unknown functional specialization of migratory and resident DC subsets, and inflammatory DCs. Cytokines specifically expressed by a given DC subset are indicated. The ability to present antigens to CD8 or CD4 T cells is represented with red or blue arrows, respectively. The ability of DC subsets to induce cytotoxic T lymphocyte (CTL) differentiation, regulatory T (Treg), or helper T (Th) cell polarization is indicated. Question marks indicate unknown functions and crosses indicate functions that are not performed by a given DC subset.

Collectively, these results suggest that some CD4 T-cell responses are the consequence of subset-intrinsic specialization, while others are more dependent on signals from the environment or on tissue imprinting (Figure 1). The clearer observations so far are the specialization of CD141^+^ DCs for Th2 polarization and of CD14^+^ DCs for Tfh polarization (both findings would need to be confirmed with DCs from other tissues), and of skin and vaginal mucosal LC and CD1a^+^ DCs for Th2 and Th22 polarization. However, this specialization might be tissue-dependent as lung CD1c^+^CD1a^+^ DCs are not potent inducers of Th2 polarization.

## Conclusion

Despite the technical challenges inherent to human DC work, significant progress has been made in the past few years in the characterization of human DC subsets. Important issues that will need further exploration include the ability of DC subsets to stimulate effector and memory T cells, the interplay between DC subsets, and the *in vivo* confirmation of functional specializations observed *ex vivo*. These could be achieved by the use of humanized mice models, the analysis of relevant pathological situations, or the study of patients with mutations in relevant genes.

This knowledge will be instrumental in the design of novel vaccines and DC-based immunotherapies.

## Conflict of Interest Statement

The authors declare that the research was conducted in the absence of any commercial or financial relationships that could be construed as a potential conflict of interest.
